# Reinforcer efficacy of grain for horses

**DOI:** 10.1002/jeab.797

**Published:** 2022-09-02

**Authors:** JoAnna Platzer, Erica N. Feuerbacher

**Affiliations:** ^1^ Department of Animal Sciences Virginia Polytechnic and State University

**Keywords:** positive reinforcement, clicker training, reinforcer efficacy, progressive ratio schedules, horse

## Abstract

Positive reinforcement is becoming more common in horse training. Identifying effective reinforcers is critical for training success. The aim of this study was to determine relative reinforcer efficacy of different grains. Four horses learned to muzzle touch a target, after which they were tested using a progressive ratio schedule of reinforcement with different grains as the consequence. Break points were used to determine relative reinforcer efficacy of each grain and were also converted into unit price per kilocalorie to determine if caloric value impacted reinforcer efficacy. Condition 1 compared three textured grains that spanned industry‐accepted standards of low to high nonstructural carbohydrate content. Condition 2 compared three pelleted grains that similarly differed in nonstructural carbohydrate content, comparable to Condition 1, but that had a different texture than those of Condition 1. Finally, Condition 3 directly compared one grain each from Conditions 1 and 2. Results showed overall little difference in reinforcer efficacy or unit price between grains but found that all grains tested functioned as reinforcers for the horses. This suggests that a range of commercially available grains can maintain behavior and therefore be used for training. We also identified possible extraexperimental factors that impact reinforcer efficacy.

Horses have lived and worked closely with humans for thousands of years and continue to do so to this day (Kelekna, 2009). Recent advances in animal behavior and welfare are changing the way that people interact with horses, including the methods chosen for teaching desirable skills to horses. Positive reinforcement training, including clicker training, has become more common as new research highlights its efficacy and benefits to horse welfare and the horse–human bond (Hendriksen et al., 2011; Innes & McBride, 2008; Sankey, Richard‐Yris, Henry et al., 2010; Sankey, Richard‐Yris, Leroy et al., 2010; Warren‐Smith & McGreevy, 2007). Horses are often required to perform long bouts of high‐effort behavior in the service of humans, so identifying positive reinforcers with greater reinforcer efficacy can help trainers maintain desirable performances.

Prior research into reinforcer efficacy for horses has explored the effectiveness of social interaction compared to food. Sankey, Henry et al. (2010) demonstrated that horses learned a task more quickly when given food as a consequence (a small piece of carrot) compared to grooming (a human scratching the horse's withers). More recently, Takahashi et al. (2016) demonstrated that horses trained to depress a target engaged in high rates of responding for a food pellet reinforcer. When the researchers switched the consequence of the target press to a neck pat, all subjects' responding dropped to nearly zero, such that the experimenters determined the reinforcing effect of a neck pat to be virtually nonexistent. When food was reintroduced as a reinforcer, responding was even higher than it was upon initial training. Kieson et al. (2020) trained horses to press signs with specific symbols to access food, a neck pat, or a neck scratch, depending on the sign chosen. Horses consistently chose the symbol for food over the social contact signs and often did not press any signs if food was not an option. Another method of assessing reinforcer efficacy is to measure break point, the highest response requirement completed before the subject fails to meet criteria, during a progressive ratio schedule. Using this method, Lee et al. (2011) found grain to be a more effective reinforcer than access to another horse or a paddock without horses. Each of the horses in this study always emitted their highest response requirement for grain: the median highest number of responses across horses was 56 for grain, 16 for access to another horse, and four for release into a paddock. In all of these studies, food was a more reinforcing stimulus than all other stimuli tested.

Other research has investigated the reinforcer efficacy of different types of food. Ninomiya et al. (2007) compared the reinforcer efficacy of two food reinforcers (grain and hay), with six horses trained with grain and six horses trained with hay. Both groups learned to engage in the response (a button press) in the same amount of time. However, when horses initially trained with hay reinforcers were switched to grain reinforcers, the number of responses emitted nearly doubled. On the other hand, when horses initially trained on grain were switched to hay reinforcers, the mean number of responses emitted decreased by over half. While their responding increased again when switched back to grain, it was still marginally lower than the initial rate of responding. Regardless of whether the horses were initially trained with grain or hay before being switched to the other food type, grain maintained more responding. Using break point data, Elia et al. (2010) similarly found that grain always had a greater reinforcer efficacy than hay, but that the reinforcer efficacy of hay and grain was sensitive to the diet the horses were fed outside of the experiment. That is, for horses maintained on hay outside of testing, grain was approximately 25 times more reinforcing than hay, while for horses maintained on grain outside of testing, hay was approximately 13 times more reinforcing than grain. This demonstrates that the extraexperimental availability of one type of food can impact the reinforcer efficacy of that type and other types of food. Taken together, these studies demonstrate that different types of food are differentially effective as reinforcers for horses, and that reinforcer value is sensitive to levels of deprivation from that food type.

A variety of stimulus features of feed might be available to horses and could impact which food is more reinforcing. Horses can associate the nutritional content of foods with organoleptic cues if they are singly presented and use this information to select foods in the future (Redgate et al., 2014). Neophobia can prevent horses from using postingestive feedback about nutritional content to make their selections (van den Berg, Lee, et al., 2016; van den Berg, Giagos, Lee, Brown, & Hinch, 2016); however, with grain (compared to forage), neophobic response was generally overcome quickly (van den Berg, Giagos, Lee, Brown et al., 2016). A study by van den Berg, Giagos, Lee, Brown et al. (2016) found that horses' preferences for grain diets were first determined by the grains' nutrient contents, then taste, then odor; similarly, Cairns et al. (2002) found that horses' individual preferences for particular flavors in grains can be overridden if the less‐preferred flavor is paired with a higher caloric content.

Given that multiple studies have found grain to be a very potent reinforcer for horses, and that these grains can vary on a multitude of dimensions that influence horse preferences, identifying if there are certain qualities of grains that impact reinforcer efficacy would be useful. Commercially available grains differ in a variety of dimensions such as ingredients, nutritional makeup, smell, taste, and texture, any of which could influence reinforcer efficacy.

This study explored the reinforcer efficacy of six commercially available grains. The grains tested varied in digestible starch and simple sugar (nonstructural carbohydrates, or NSC; King & Mansmann, 2004) content. Eating high‐NSC grain has been linked to a number of digestive and metabolic disorders in horses, including colic, laminitis and chronic founder, developmental orthopedic disease, Cushing's disease, and obesity (Hoffman, 2009). It has also been linked to increased behavioral and physiological reactivity, likely via changes in microbiota (Bulmer et al., 2015; Bulmer et al., 2019). Observers rated horses fed a high NSC diet to be more nervous/tense/unsure (Bulmer et al., 2019), and horses were more likely to display a fear response (blowing) in response to a novel object (Destrez et al., 2019). Young horses fed lower NSC diets were less distressed after weaning and seemed calmer, less fearful, and more inquisitive during temperament tests than those fed higher NSC diets (Nicol et al., 2005). For these reasons, NSC (colloquially called “starch”) can be a factor that horse owners consider when deciding which grains to feed their horses (Moore, 2018) and the grains in this study were selected specifically to vary on their percentage of NSC.

To compare the reinforcer efficacy of these grains, the horses in this study were reinforced on a progressive ratio schedule, in which they were required to emit successively more responses (increasing response requirement) to access reinforcement across trials within a session. Progressive ratio schedules have been used to successfully investigate the reinforcer efficacy of a variety of stimuli (grain vs. cribbing, Houpt, 2012; grain vs. access to a paddock or a conspecific, Lee et al., 2011; hay vs. grain, Elia et al., 2010) for horses. This experiment used break point, the highest response requirement completed before the horse failed to respond within the time allotted, as its measure of reinforcer efficacy, with higher break points indicating higher reinforcer efficacy.

## Method

### Subjects

Four thoroughbred geldings (*Equus caballus*) between the ages of 8 and 11 participated in this study (Bobcat, Flyer, Roach, and Red). The horses were not ridden and had no exercise requirements. They lived outside, rotating between three pastures. Pastures ranged in size from 56 m x 57 m to 87 m x 72 m, measured by Google Maps. Each pasture contained round hay bales, a lean‐to shed, and an automatic waterer. Each pasture housed three to four horses at a time and was adjacent to other pastures that contained horses. The use of the horses was in accordance with the regulations of Virginia Tech's Institutional Animal Care and Use Committee.

### Training

#### Setting

Training occurred with the horse loose inside a round pen made of metal corral panels that was within sight of their pasture‐mates to limit stress from separation. Experimenters remained outside the round pen.

#### Reinforcer

Horses received pre‐portioned servings of approximately 5.2 g of SafeChoice Special Care (Table 1) grain as reinforcers during training.

**Table 1:** Grain Characteristics

Brand Name of Grain	Study Name of Grain	% NSC	% Protein	% Fat	Kilocalories Per Serving	Weight Per Serving (g)
Grains – Condition 1						
ProElite Senior	LT	12.9	14	10	12.8	4.0
Legends Growth	MT	20	14	6	13.6	4.4
Legends Sport Horse	HT	41	12	6	13.6	4.4
Grains – Condition 2						
SafeChoice Special Care	LP	13	14	7	14.6	5.2
SafeChoice Maintenance	MP	25	14	5	15.1	5.6
Triumph Professional	HP	34	12	8	18.6	5.9
Grains – Condition 3						
ProElite Senior	LT	12.9	14	10	12.8	4.0
SafeChoice Special Care	LP	13	14	7	14.6	5.2

#### Training Procedure

Before the target was introduced, the experimenters paired the marker, a click from a mechanical clicker (Good2Go soft click), with the primary reinforcer five times by delivering a serving of grain immediately following a click. Bobcat startled at the sound of the clicker, so for him a verbal “yes” was used in place of a click (within this manuscript any mention of a “click” will mean a verbal “yes” for Bobcat). Following this, the target was introduced. The target was a Styrofoam ball (15 cm in diameter) on a wooden dowel (20 mm in diameter, 1 m long). Red duct tape covered the ball and extended 15 cm down the dowel and a clicker was affixed to the dowel's base so that the experimenter could easily click while holding the target stick. The click was used to mark that a response requirement had been met—it immediately followed a response and preceded reinforcement.

Over several short (<5 min) sessions on different days, the horses learned to touch their muzzle, defined as the area of the horse's face below where the halter sat, to the target via shaping. First, the experimenter presented the target approximately 0.25 m from the horse's face. Once the horse touched the target with his muzzle, the experimenter immediately clicked, removed the target, and delivered a serving of grain on a flat palm. Once the horse finished chewing, the target stick was re‐presented. The experimenter repeated this process, gradually increasing the distance the target was presented from the horse. If the horse failed to touch the target within approximately 30 seconds, the target was re‐presented at a closer distance until the horse was predictably touching the target at that distance, after which distance was increased once again.

After the horses had met the response requirement approximately 45 times and would readily touch the target presented 1 m away in the round pen, they were evaluated to see if they would emit the target response in the testing setting. Testing occurred in a stall instead of the round pen because grass, a competing reinforcer, grew in the round pen, and the availability of grass could not be standardized between sessions. Horses had to complete 20 target touches on a fixed ratio (FR) 1 schedule in the testing stall without more than 30 s elapsing between touches before they could begin testing. All horses in this study met this criterion on the first assessment.

### Testing

#### Setting

The wooden stall was approximately 3.4 m by 3.5 m with screened‐in windows on two sides. Three sides of the stall had walls approximately 3.5 m high but the remaining side connected to an adjacent stall by a half‐wall over which the horses could extend their heads. The half‐wall was comprised of solid wood approximately 1.2 m tall and three wooden planks above that through which the experimenter could extend their hand to deliver grain. The stall had rubber flooring covered with sawdust, a filled five‐gallon water bucket, and an empty feed bucket. During testing, the experimenter and assistant(s) remained in the adjacent stall. The experimenter remained approximately 0.3 m from the half‐wall and the assistant sat in a chair approximately 1.5 m back from the half‐wall. For the trials in which a second assistant was present to gather interobserver agreement, the second assistant sat in a chair beside the first assistant.

#### Reinforcers

To ensure a standardized amount of grain could be delivered with minimal delay, servings of grain used as reinforcers were always pre‐portioned into individual paper cups prior to testing. Servings of all grains tested measured approximately 3.75 mL by volume using a graduated medicine cup. The mean weight in grams of 50 servings was taken to represent the weight of a single serving for analysis (Table 1).

#### Testing Procedure

**Figure 1:**
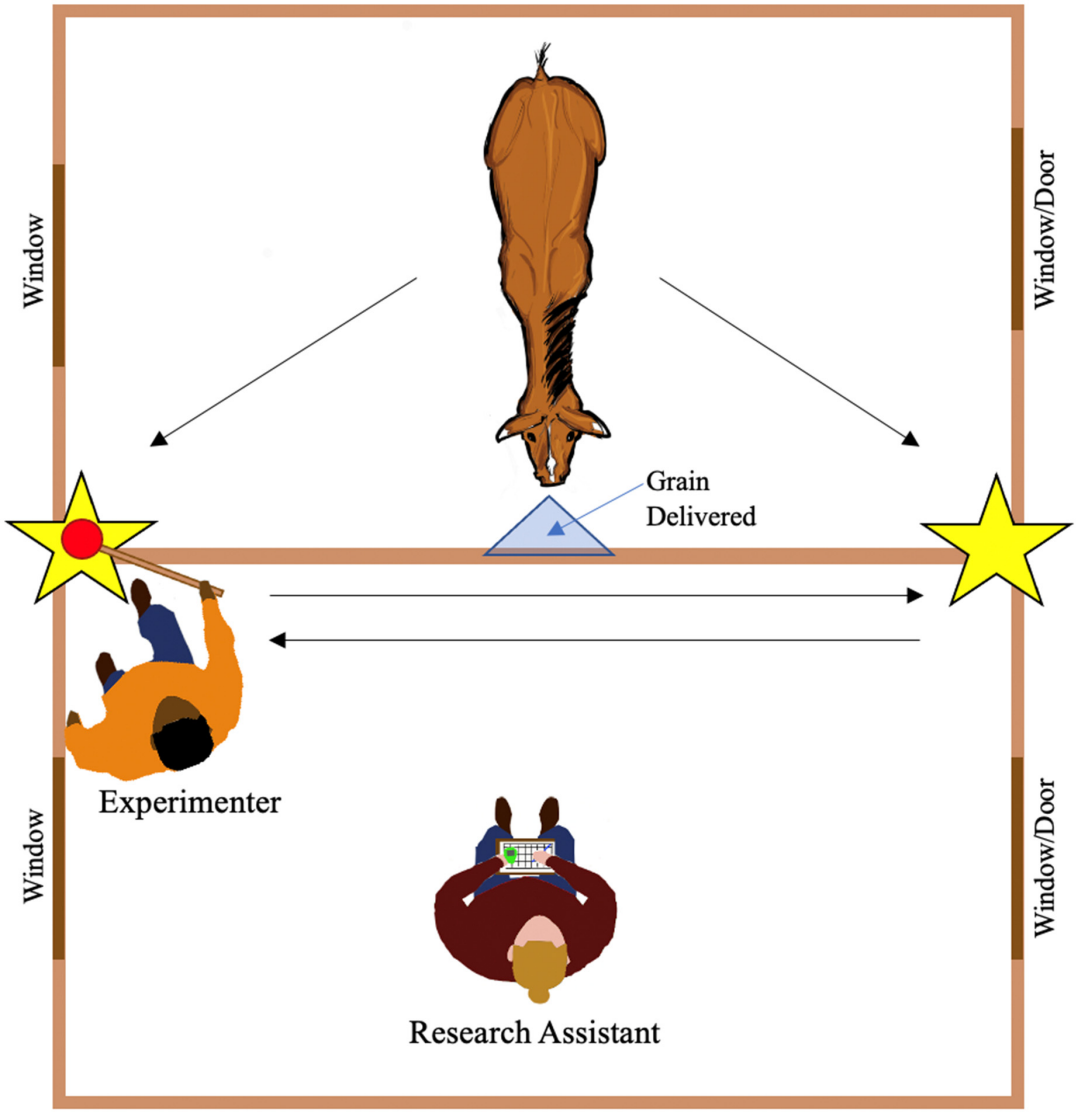
*Aerial View of the Testing and Adjacent Stall* *Note*: The horse remained in the testing stall and the experimenter and assistant(s) in the adjacent stall. The experimenter presented the target at the locations marked by stars.

Horses were always tested in the afternoon so that they had been deprived of grain for at least 3 hr. One horse was tested at a time. Once the experimenter brought the horse into the testing stall from the pasture, unclipped their lead rope, and moved to the adjacent stall, testing began immediately. Using the same target used in training, the experimenter placed the target in the corners of the stall where the half‐wall met the full wall on either side, approximately 1.7 m off the ground (marked by stars in Fig. 1). The experimenter faced the half‐wall and did not make eye contact with or talk to the horse. As with training, the target response was a muzzle touch to the target, though during testing the horse had to walk back and forth across the testing stall to access the target. Horses worked on a Basis 2 Progressive Ratio 1 schedule (Feuerbacher et al., 2022; Vicars et al., 2014), such that the horse had to complete each response requirement twice before the response requirement was increased by one touch. Response requirements began at one touch at the beginning of each testing session.

When the horse completed a touch that was not the final touch in the response requirement for that trial, the experimenter immediately removed the target and walked to the opposite corner of the half‐wall to re‐present the target in the other corner, repeating this until the response requirement for that trial was complete or the horse timed out (1 min elapsed from the presentation of the target without the horse touching it). If the horse timed out, the assistant said “time” and the experimenter immediately removed the target and returned the horse to his pasture.

When the horse completed the current response requirement, the experimenter immediately clicked, removed the target, and delivered a pre‐portioned serving of grain on a flat palm over the half‐wall at the midpoint between the two target placement locations (Fig. 1). Once the horse had taken the grain, the experimenter would immediately start the next response requirement in the sequence, alternating the side on which the target originated so that it was presented in both corners equally.

#### Conditions

This experiment consists of three conditions, each comparing a different combination of grains. They are listed in chronological order.

##### Condition 1

Condition 1 compared three textured grains that varied in macronutrient and caloric content (Table 1). These grains represented the greatest extremes of NSC content the experimenters found on the market while maintaining a common texture (textured) to minimize differences in palatability. The same company (Cargill) manufactured all three grains. LT was the low‐NSC grain, MT the medium‐NSC grain, and HT the high‐NSC grain. Three horses (Bobcat, Flyer, and Red) had equal exposure to each of these grains in a pilot study for this experiment, one month prior. Roach did not participate in the pilot study, so this was his first contact with these grains.

All horses were tested on all three grain types, but only one grain was delivered in a given session, and only one session was conducted per day (i.e., only one grain tested per day). Horses were first tested on LT, followed by MT, and then HT. After a horse had been tested on all three grain types, the sequence started again. Testing days were not always contiguous but sessions within a given low‐medium‐high cycle never occurred more than two days apart. Each horse was tested on each of the three grains four times, for a total of 12 sessions.

Throughout Condition 1, the horses were maintained on daily rations of 1.36 kg of textured grain per horse and grass hay available *ad libitum* except when in the testing stall. Horses received their daily rations once in the morning in their pastures, placed in individual pans approximately 5 m apart. Their staple grain was custom formulated by a local mill and comprised 26.9% NSC, 17.7% protein, and 9.9% fat with 3.21 Mcals/kg digestible energy.

##### Condition 2

Condition 2 replicated Condition 1 but with grains of a different texture (pelleted) to further investigate the effect of NSC content on reinforcer efficacy. Condition 2 compared three pelleted grains that varied in macronutrient and caloric content (Table 1). Like Condition 1, these grains varied in NSC content while maintaining a common texture (pelleted). The same company (Cargill) manufactured all three grains. LP was the low‐NSC grain, MP the medium‐NSC grain, and HP the high‐NSC grain. All horses had equal exposure to LP during the training phase, several months prior.

Just as in Condition 1, all horses were tested on all three grain types, but only one grain was delivered in a given session, and only one session was conducted per day. Horses were first tested on LP, followed by MP, and then HP. After a horse had been tested on all three grain types, the sequence started again. Testing days were not always contiguous but sessions within a given low‐medium‐high cycle never occurred more than 3 days apart. Each horse was tested on each of the three grains four times, for a total of 12 sessions.

Feeding outside of testing remained the same as in Condition 1.

##### Condition 3

Condition 3 directly compared one grain from each of the prior conditions (Table 1). While Conditions 1 and 2 compared grains similar in texture (textured, Condition 1; pelleted, Condition 2) but varying in NSC content, Condition 3 explicitly explored the effect of grain texture on reinforcer efficacy.

Just as in Conditions 1 and 2, all horses were tested on both grain types, but only one grain was delivered in a given session, and only one session was conducted per day. However, in Condition 3, the order in which the grains were tested was randomly counterbalanced, with two horses starting with LT and two starting with LP. On subsequent sessions, horses alternated between the two grains across testing days. Testing days were not always contiguous but never more than 2 days apart. Each horse was tested on each of the two grains seven times, for a total of 14 sessions.

Feeding outside of testing remained the same as in Conditions 1 and 2 except for grain rations. Due to a change in facility policy, before Condition 3 the horses' staple grain switched from textured to pelleted form and the total amount and feeding schedule of grain changed. Throughout Condition 3 the horses received an additional 0.45 kg of grain in their daily ration, for a total of 1.81 kg. Half of the 1.81 kg was fed in the morning and the other half in the evening, after testing finished. The nutritional makeup of the grain did not change.

#### Analysis

The assistant timed and recorded the highest response requirement completed by the horse in each session (break point). For 25% of sessions, a second assistant also timed and recorded the highest response requirements completed by each horse for interobserver agreement. Interobserver agreement across conditions for highest response requirement completed was 100%.

The multielement design data were graphed on a session‐by‐session basis to visually analyze each grain's reinforcer efficacy performance in each condition. Visual analysis was supported by statistical analysis using Tau‐U (Parker et al., 2011). It was calculated using Single Case Research's online software (http://singlecaseresearch.org/calculators/tau-u). All other statistics were calculated using GraphPad Prism 8.

In addition to using break points to evaluate the reinforcer efficacy of the grains as a whole, the unit price per kilocalorie for each grain was calculated. Cargill nutritionists provided kilocalories per gram for each grain, which was used to convert break points to unit price (responses/kilocalorie of grain received) to evaluate whether caloric content varied with responding.

## Results and Discussion

### Condition 1

**Figure 2:**
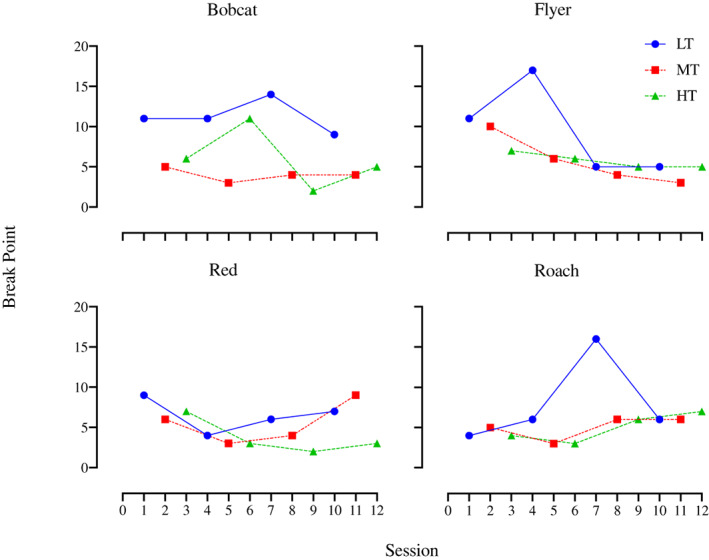
Condition 1 Break Points Per Horse Across Sessions

Condition 1 compared three textured grains varying in NSC content. Figure 2 shows the session‐by‐session break points for each horse in Condition 1. Only one horse (Bobcat) showed a clear separation of data paths via visual and statistical analysis (LT garnered more responding than MT and HT, Tau ‐0.88, *p* = .004), which suggests that within this condition, LT was the most efficacious reinforcer for him (highest break point: 14). Though LT did not show the same pattern with the other horses, it tended to have the highest break point within a given LT‐MT‐HT cycle, and it had the single highest break point for Flyer (17) and Roach (16) and tied for highest with MT for Red (9).

**Figure 3:**
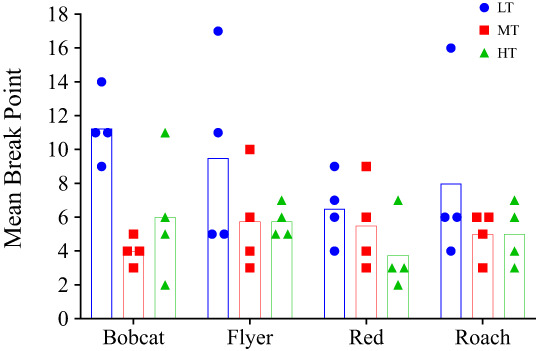
*Condition 1 Mean Break Points Per Horse* *Note*: Bars represent mean break points and symbols represent individual break points.

Supporting the session‐by‐session data, all horses had their highest mean break point for LT (Fig. 3). There was no clear pattern between MT and HT: Bobcat had his second highest mean break point for HT and Red had his second highest mean break point for MT, while MT and HT were equal for both Flyer and Roach. The mean break points for all horses together were 8.8 for LT, 5.1 for MT, and 5.1 for HT. A Friedman's test found that the mean break points for LT were significantly greater than MT and HT (*X* ^*2*^ = 6.86, *p* = .037) but a Wilcoxon Signed Rank Test found that LT was not significantly greater than either MT (*Z* = 1.15, *p* = .125, *r* = 0.58) or HT (*Z* = 1.15, *p* = .125, *r* = 0.58).

### Condition 2

**Figure 4:**
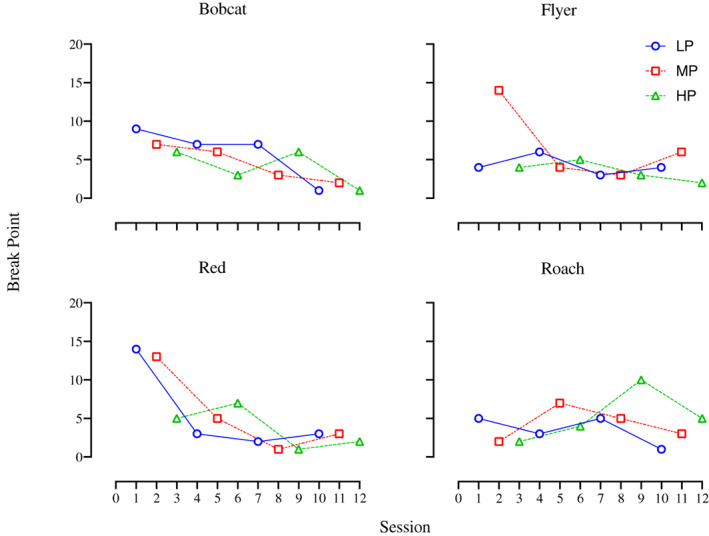
Condition 2 Break Points Per Horse Across Sessions

To further explore if low‐NSC content predicted higher reinforcer efficacy, Condition 2 compared three pelleted grains similarly varying in NSC content but with a different texture than the grains in Condition 1. Figure 4 shows the session‐by‐session break points for each horse. For all horses, the data paths overlap considerably, suggesting that one grain was not more reinforcing than the others. Bobcat, who showed the most differential responding in Condition 1, also showed the most differentiation here, with LP having the highest break points for three cycles (highest break point, 9). Flyer, Red, and Roach had their single highest break point for MP (14), LP (14), and HP (10), respectively, with substantial overlap in their data paths such that visual or statistical analysis was not able to determine a pattern of reinforcer efficacy across grains.

**Figure 5:**
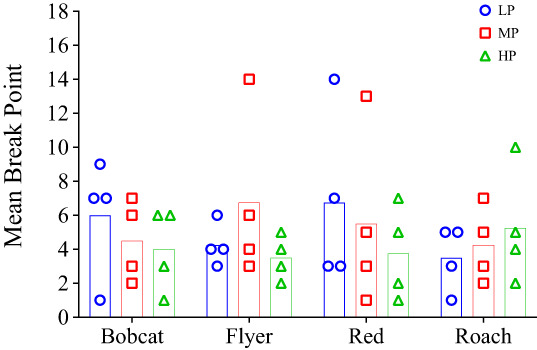
*Condition 2 Mean Break Points Per Horse* *Note*. Bars represent mean break points and symbols represent individual break points.

Paralleling the session‐by‐session data, the mean break points per horse in Condition 2 varied across horses and no grain consistently maintained more responding (Fig. 5). Bobcat and Red had their highest mean break point for LP (6 and 5.5, respectively), Flyer for MP (6.8), and Roach for HP (5.3). Despite each grain producing the highest break point for at least one horse, the differences in mean break points between grains for each horse were relatively slight with considerable variance in individual data points, and mean break points overall were similar across horses. Thus, no grain appeared to function as a more valuable reinforcer than the others either for an individual horse or overall. The mean break points for all horses together were 4.8 for LP, 5.3 for MP, and 4.1 for HP. Supporting the visual analysis, statistical analysis did not find that the grains differed significantly (Friedman's test: *X* ^*2*^ = 1.73, *p* = .53).

### Caloric Content

**Figure 6:**
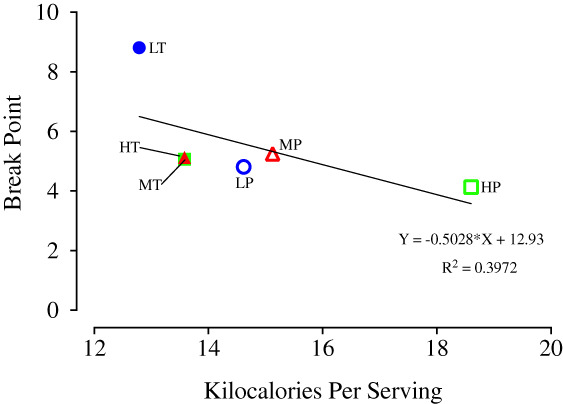
Caloric Content Versus Break Points of Grains from Conditions 1 and 2

Though Conditions 1 and 2 were designed to explore the impacts of NSC content on reinforcer efficacy, the grains tested in these conditions also varied in caloric content (Table 1). Prior research suggests that horses can detect and associate nutritional content, including caloric content, with the foods they ingest, including processed grain (Cairns et al., 2002). To explore the possible relationship between caloric content and reinforcer efficacy, the caloric content of all six grains used in Conditions 1 and 2 were graphed against their mean break points (Fig. 6). A simple linear regression found a negative relationship (Y = ‐0.5*X + 12.93; *p* = .18; ƒ^2^ = 0.66) between caloric content and mean break point (Fig. 6). The cost (amount of responding) the horses paid for grains tended to decrease as the caloric content of the grains increased. This contrasts with Cairns et al. (2002) that found that horses would preferentially select a higher calorie grain in a concurrent choice preference assessment.

One possible explanation for the observed trend is that, within sessions, horses may satiate more quickly on a higher calorie grain and thus emit fewer responses overall than they would for lower calorie grains (DeGrandpre et al., 1993). If this were the case, this would decrease break points in higher calorie grains and inflate the apparent reinforcer efficacy of lower calorie grains relative to higher calorie grains. In terms of physiological satiation, horses consumed considerably more grain during their daily rations than they did during testing. During Conditions 1 and 2, horses consumed a mean of 51.4 g and 50.4 g, respectively, per session. In contrast, the horses consumed 1360 g of grain within a matter of minutes during their daily rations, meaning that horses were only receiving approximately 3.7% of the amount they would willingly consume during mealtime. This suggests that horses did not stop responding because of a physical satiation. However, horses consumed approximately equal mean kilocalories per session: 161.2 kcal (3.7% of daily intake) and 145.0 kcal (3.3% of daily intake) for Conditions 1 and 2, respectively. If the horses were regulating their caloric consumption against the cost of obtaining the grains, this would account for the lower break points in Condition 2 (which had more calorie‐dense grains; Table 1).

### Condition 3

Condition 3 directly compared one grain, matched for NSC content but opposite in texture, from each of the previous two conditions against each other. LT was selected to see if it maintained its reinforcer efficacy advantage when tested against a grain matched for NSC content, LP, to eliminate the possibility of contrast effects due to varying NSC content and further explore the role of grain texture in reinforcer efficacy.

**Figure 7:**
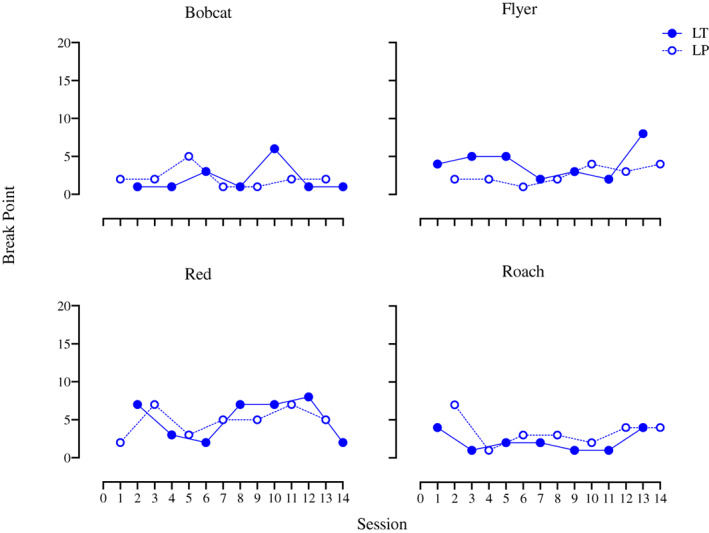
Condition 3 Break Points Per Horse Across Sessions

Figure 7 shows the session‐by‐session break points for each horse for Condition 3. There was a range of individual variation between horses but the differences between grains per horse were relatively small. For Bobcat, LP always had equal or higher break points than LT in an LP–LT pair of sessions with one exception (Session 10), which happened to be his single highest break point (6). Roach, showing the opposite pattern, was the most consistent: LP was always equal to or higher than LT within a LT–LP pair of sessions. His single highest break point was for LP (7). For Flyer, the break points for LT were always equal to or higher than LP within LT–LP pairs of sessions with two exceptions (Sessions 10 and 12) immediately prior to his highest break point (LT, 8, Session 13). Red was the most variable, showing extensive overlap between data paths; his highest break point was with LT (8). Overall, both grains performed similarly for all horses. Statistical analysis of the results from Condition 3 likewise did not find any significant differences in the reinforcer efficacy of these two grains for any horses, however, LT in Condition 3 had significantly lower break points than it did in Condition 1 for Bobcat (Tau ‐1, *p* = .008) and Roach (Tau ‐.93, *p* = .014).

**Figure 8:**
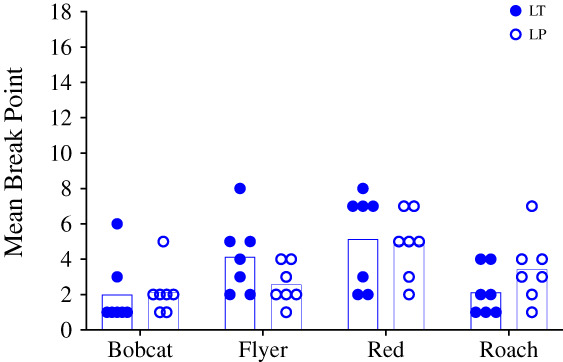
*Condition 3 Mean Break Points Per Horse* *Note*: Bars represent mean break points and symbols represent individual break points.

Echoing the session‐by‐session data, the mean break points for both grains were fairly close together for each horse and there was variation between horses, indicating that the grains performed similarly in terms of reinforcer efficacy across horses (Fig. 8). Flyer and Red had higher mean break points for LT, while Bobcat and Roach had higher mean break points for LP, but overall the means were quite similar (3.4 for LT and 3.3 for LP). A Wilcoxon's Matched Pairs Signed Rank test supported the visual interpretation that the grains did not differ significantly (*Z* = 1.17, *p* = .88, *r* = 0.59).

### Unit Price

For all conditions, the unit price per kilocalorie (Table 2) was also calculated. This unit of measure reflects the cost per calorie that the horses paid in muzzle touches for each grain. Unit price is a useful measure as it combines the amount of responding and the amount of reinforcement into one variable, which can more effectively predict the behavioral effects of manipulating either schedule of reinforcement or magnitude of reinforcement (in this case, caloric content of grain) than considering either of those variables alone (DeGrandpre et al., 1993). The unit price of LT decreased from 0.7 to 0.3 from Condition 1 to Condition 3 and the unit price of LP decreased from 0.3 to 0.2 from Condition 2 to Condition 3.

**Table 2:** Mean Break Points (BP) and Unit Price (UP) per Kilocalorie

		Bobcat		Flyer		Red		Roach		Mean	
Condition	Grain	BP	UP	BP	UP	BP	UP	BP	UP	BP	UP
1	LT	11.3	0.9	9.5	0.7	6.5	0.5	8.0	0.6	8.8	0.7
1	MT	4.0	0.3	5.8	0.4	5.5	0.4	5.0	0.4	5.1	0.4
1	HT	6.0	0.4	5.8	0.4	3.8	0.3	5.0	0.4	5.1	0.4
2	LP	6.0	0.4	4.3	0.3	5.5	0.4	3.5	0.2	4.8	0.3
2	MP	4.5	0.3	6.8	0.4	5.5	0.4	4.3	0.3	5.3	0.3
2	HP	4.0	0.2	3.5	0.2	3.8	0.2	5.3	0.3	4.1	0.2
3	LT	2.0	0.2	4.1	0.3	5.1	0.4	2.1	0.2	3.4	0.3
3	LP	2.1	0.1	2.6	0.2	4.9	0.3	3.4	0.2	3.3	0.2

*Note*: Mean break point (BP) and unit price (UP) per kilocalorie provided for each grain in each condition.

## General Discussion

All grains were reinforcing to all horses in all conditions, but responding varied between horses and across conditions. Condition 1 compared three textured grains that varied in NSC content, and the findings suggested that the low‐NSC grain had the highest reinforcer efficacy for all horses. Compared to the other conditions, Condition 1 had the highest break points overall and showed the most differentiation between grains (Table 2). Condition 2 tested three pelleted grains spanning NSC content similar to Condition 1, but all Condition 2 grains produced similar break points, meaning that Condition 2 findings did not support the hypothesis that low‐NSC content predicts higher reinforcer efficacy. The break points for Condition 2 were also generally lower than those in Condition 1 (Table 2). To explore if this was due to some facet of the grains themselves, such as texture or caloric content, or perhaps due to some extraexperimental factor, Condition 3 tested one grain from each of the prior conditions, matched for low‐NSC content but differing in texture, directly against each other.

In Condition 3, the grains had very similar reinforcer efficacy, with almost identical mean break points for all horses together (Table 2). Condition 3 showed the least differentiation between grains out of all conditions, despite LT's mean break point being almost double that of LP's in their respective prior conditions (Table 2). Condition 3 also had the lowest break points of all conditions, with both grains' mean break points decreasing considerably in Condition 3 relative to prior conditions (Table 2). Variation in grain performance could be a result of extraexperimental factors impacting the horses' responding.

One extraexperimental factor that was not controlled for was time of year. Condition 1 was conducted in the summer, Condition 2 in the winter, and Condition 3 in the spring, so it is possible that variables associated with time of year, such as weather or the quality and availability of forage, may have impacted responding. Another extraexperimental factor that may have impacted responding is the grain the horses received during their daily rations. Elia et al. (2010) found that feeding horses grain as their staple food outside of testing decreased the reinforcer efficacy of grain during testing. During Condition 3 horses were fed 33% more grain during their daily rations outside of testing relative to earlier conditions. The increased access to grain could have functioned as an abolishing operation diminishing the horses' responding for access to grain. During Condition 3, horses received a mean of 84.1 kcals or 28.2 g of grain per session; this was 1.5% and 1.6% of the kilocalories and grams of grain, respectively, that they received in their daily ration. This is less than half the percentages they received during the prior conditions, suggesting that as calories or access to a specific food are more widely available to them, the cost (amount of responding) that the horses would pay may decrease. Even with increased extraexperimental access, the grains still functioned as reinforcers for the horses, and could still be used to reinforce low‐effort tasks.

The current study does present some limitations beyond the extraexperimental factors. Most notably, when comparing different foods, it is nearly impossible to vary one factor, such as caloric content, without altering another factor, such as ingredients. The grains in this study were no exception: They varied multidimensionally, so definitively linking a certain facet of the grain, such as NSC content, to reinforcer efficacy would require additional experimentation, manipulating other factors that differ across grains. Additionally, the order in which the grains were tested could have resulted in contrast effects impacting results, as Conditions 1 and 2 were not counterbalanced. However, one horse from each counterbalanced pair in Condition 3 had higher break points for each grain tested, suggesting that the order in which the grains were tested did not considerably affect reinforcer efficacy. Lastly, the chosen behavior was arbitrary and repetitive, and the experimental conditions may not have been reflective of real‐life training scenarios. Future research is needed to examine how the multitude of variables that differ between foods can impact the efficacy of food reinforcers, how extraexperimental factors such as what horses eat outside of training can impact responding, and how grains perform as reinforcers in more naturalistic applied training scenarios.

The results of this experiment can be practically related to horse training. All grains tested were reinforcing to all horses tested, highlighting its utility as a training tool. Responding was variable, but at its best, a horse chose to walk back and forth across a stall to touch a target seventeen times for about a pinch of grain. Grain is relatively easy to deliver, widely available, economical, and already a part of many horses' diets. The results of this study suggest that a wide variety of grains that differ in texture, ingredients, nutritional makeup, and caloric content would function as reinforcers, so it is likely that a trainer could find a grain suitable to their horse's dietary needs that could be used for training. Relative to other reinforcers, grain is likely more reinforcing than hay (Elia et al., 2010), but further research is needed to compare its reinforcer efficacy to other foods such as commercial horse treats or carrots, which have also been shown to have higher reinforcer efficacy than hay for some horses (Fox & Belding, 2015).

As the horses in this study displayed individual differences in which grains functioned as more effective reinforcers, trainers may benefit from conducting a reinforcer efficacy assessment for each horse they will be training. Though additional research needs to further explore the trends observed in this study, it is possible that textured grain texture, lower calorie, and lower NSC content may correlate with greater reinforcer efficacy or at least fail to negatively impact it. Moreover, unless a trainer has reasons for choosing otherwise, it may be advisable to preferentially select a grain lower in calories, to decrease chances of satiation or unintentional weight gain, and lower in NSC (“starch”), to decrease the possibility of the health and behavioral concerns associated with feeding a higher‐NSC food (e.g., Bulmer et al., 2015; Bulmer et al., 2019; Hoffman, 2009). Additionally, eating a high‐NSC diet has been linked to horses spending less time standing and investigating their environment and more time in a heightened alert state (Bulmer et al., 2019), which could negatively impact learning (McLean & Christensen, 2017) and cause unnecessary difficulties during training.

Given prior research findings (Elia et al., 2010) and the possible extraexperimental effects observed in this experiment, trainers may benefit from using a different grain or even different food reinforcers in training than the horse is fed during their daily rations; if this is prohibitive, however, the results of this study suggest that using a portion of the horse's daily rations for training will likely still be successful if the horse is not satiated on it. Trainers should consider and monitor their horse's caloric and nutritional intake closely, as what horses receive for rations can impact training and what horses receive during training should likely impact what they receive for rations to maintain ideal health and wellness.
